# Prevalence of *Helicobacter pylori* cagA virulence factor and validation of serological tests in a population from Northeastern Brazil

**DOI:** 10.1590/1414-431X2025e14906

**Published:** 2025-10-17

**Authors:** A.C. Freitas, I.A. Santana-Santos, I.S. Lima, D.K. Queiroz-Santos-Trindade, L.F. Sandes, T.M.L. Correia, D.B. Almeida, M.A. Lescano-Lescano, L.C. Santana, C.A. Figueiredo, H.S. Silva, L.C. de Lima, F.F. de Melo, T.M. da Silva, C.R. Marques

**Affiliations:** 1 Instituto Multidisciplinar de Saúde da Universidade Federal da Bahia, Vitória da Conquista, BA, Brasil; 2 Departamento de Ciências Biológicas, Universidade Estadual do Sudoeste da Bahia, Vitória da Conquista, BA, Brasil; 3 Departamento de Ciências da Saúde, Universidade Estadual do Sudoeste da Bahia, Vitória da Conquista, BA, Brasil; 4 IBR Hospital, Vitória da Conquista, BA, Brasil; 5 Instituto de Ciências da Saúde, Universidade Federal da Bahia, Salvador, BA, Brasil

**Keywords:** ELISA, Serology, Sensitivity, Virulence, Helicobacter pylori

## Abstract

*Helicobacter pylori* is an infectious agent linked to significant gastric pathologies, which makes it a public health concern. The enzyme-linked immunosorbent assay (ELISA) is widely used for epidemiological studies and for investigating virulence factors like the *cagA* gene. Due to the varying antigenic profiles of bacterial strains across different populations, the local validation of serological tests is essential. This study aimed to evaluate the performance of two commercial serological tests - the MyBiosource HP-CagA-IgG ELISA kit and the Sunlong Human IgG (CagA-IgG) ELISA kit - in detecting the cagA virulence factor and to assess its prevalence in bacterial isolates from a population in the southwest region of Bahia. A total of 88 individuals were enrolled, and 34 tested positive for the cagA factor via real-time PCR. After establishing customized cutoff points, the MyBiosource kit demonstrated a sensitivity of 55.88%, specificity of 50%, and accuracy of 52.22%, while the Sunlong kit showed a sensitivity of 70.59%, specificity of 60%, and accuracy of 64.29%. Despite these results, neither test met satisfactory performance standards, with sensitivity below 75% and specificity ranging from 50 to 60%. The overall prevalence of *H. pylori* infection was 56.8%, with a cagA prevalence of 68% among positive cases. Further investigations using additional commercial tests are recommended to enhance diagnostic outcomes for this population.

## Introduction

*Helicobacter pylori* is a spiral-shaped, gram-negative, flagellated microaerophilic bacillus that induces hypochlorhydria in the gastric mucosa. This bacterium is widely recognized as one of the primary etiological agents of gastric diseases, including gastritis, peptic ulcers, lymphoid tissue lymphoma, and gastric cancer. Its pathogenicity and association with these conditions underscore the importance of understanding its mechanisms and developing effective diagnostic and therapeutic strategies (1). Approximately half of the global population is estimated to be infected with *H. pylori* (2), with prevalence notably high in developing regions, particularly in South American and African countries such as Brazil, Chile, Ethiopia, and Nigeria. Estimates indicate that between 70 and 90% of the population in these areas is affected, with the highest rates observed in economically disadvantaged communities (3,4). In contrast, in developed countries such as Australia, the United States, and Switzerland, the prevalence does not exceed 30% (2).

Transmission of *H. pylori* primarily occurs during childhood, with the principal routes of contamination being gastro-oral, oral-oral, and oral-fecal. The infection can be transmitted through contaminated water, food, saliva, vomit, and feces (1). Virulence factors encoded by *H. pylori* genes are associated to enhanced pathogenicity and the exacerbation of gastric diseases (5). The cag pathogenicity island (cag-PAI) is a genetic region present in certain strains of *H. pylori.* This region demonstrates significant genetic variability and is closely associated with increased virulence and more severe disease manifestations (6,7). Among the virulence genes identified within the cag-PAI, the *cagA* oncogene (cytotoxin-associated gene A) has been extensively investigated for its capacity to disrupt cellular pathways, ultimately contributing to the progression of gastric carcinoma (8,9).

*H. pylori* infection can be diagnosed using invasive/minimally invasive and non-invasive methods (10). Among the non-invasive approaches, serological tests employing the enzyme-linked immunosorbent assay (ELISA) are particularly common. One of the primary advantages of the ELISA is its capacity to facilitate the investigation of virulence factors associated with bacteria (11). These tests are also favored in epidemiological studies due to their cost-effectiveness, rapid turnaround time, and high reproducibility (12). Furthermore, considerable diversity exists in the antigenic profiles of *H. pylori* strains circulating within populations in the same region (7,11).

Given the considerable diversity in the antigenic profiles of *H. pylori* strains within the same region, the performance of diagnostic tests can vary. To address this, our study aimed to evaluate serological kits for detecting the cagA factor and to establish a new cutoff point to optimize sensitivity, specificity, positive predictive value, and negative predictive value (6). This local validation is essential for ensuring the effectiveness of these tests in both diagnostic and screening contexts within epidemiological studies.

## Material and Methods

### Study population and biological samples collection

Eighty-eight individuals aged 16 years and older who underwent upper gastrointestinal endoscopy (EDA) were recruited for this study between April 2019 and December 2022. Samples were collected at the General Hospital of Vitória da Conquista (HGVC) and at the Digestive System Institute (IAD) in Vitória da Conquista, southwest Bahia, prior to the gastroduodenal endoscopy procedure, mostly in the morning. The following exclusion criteria were established: pregnant or lactating women; individuals who had previously undergone treatment for *H. pylori* eradication; those who had used antibiotics within 30 days before the examination or proton pump inhibitors within 14 days preceding the procedure; individuals with systemic arterial hypertension, coagulation disorders, or any anatomical condition that would obstruct endoscopy; immunocompromised patients; and those with a history of gastric surgery.

The sample size was based on observations from previous studies (11,13). A total of 70 patients (35 *H. pylori* positive) were required, assuming a prevalence of 50% and an assay sensitivity of 90%, with a margin of error of 10%. Participants completed a standardized questionnaire covering individual information, socio-demographic and economic characteristics, living conditions, and sanitation. All study procedures were approved by the Research Ethics Committee of the Universidade Estadual do Sudoeste da Bahia (UESB), with approval number 2.450.642. The informed consent form and assent form were signed by all participants. The study was conducted following the guidelines outlined in the Declaration of Helsinki.

### Sample collection and processing

During the upper gastrointestinal endoscopy, tissue samples were obtained from the antrum's lower curvature and the gastric body greater curvature. These samples were preserved in a medium with Brucella agar, 30% glycerin, and sterile water. They were subsequently used for *H. pylori* diagnosis through rapid urease testing, DNA extraction, and bacteriological culture. Additionally, peripheral blood samples were collected for serological testing. Blood was drawn into serum tubes containing a clot activator gel and stored at 5°C until laboratory processing. The blood samples were then centrifuged at 2500 *g* for 10 min at 4°C. After centrifugation, the serum was aliquoted into Eppendorf tubes and stored at -20°C until needed for serological testing.

### Bacteriological culture

Gastric mucosa fragments collected for culture were stored in BHI transport medium (Brain Heart Infusion Broth, Difco, USA) at 4°C until they were sent for processing in the laboratory. The fragments were plated on BHI medium and bacteriological medium, supplemented with 5 mL of 10% sheep blood, 500 mL of vancomycin + nalidixic acid (Sigma Chemical Co., USA), 250 mL of cycloheximide, and 250 mL of triphenyl tetrazolium chloride (Reagen, Brazil). The plates were identified, dated, and incubated at 37°C under a microaerophilic atmosphere using Anaerocult C^®^ (Merck, Germany) and analyzed on the 3rd, 7th, and 11th days of incubation.

### Molecular analysis

*H. pylori* DNA was extracted from gastric biopsy samples using the PureLink™ kit (Invitrogen^®^, EUA) according to the manufacturer's instructions. The specific 16S rRNA and *ureA* genes in the bacteria were amplified using polymerase chain reaction (PCR) by Taqman technology.

To investigate the cagA factor of *H. pylori*, DNA from biopsy samples was amplified using real-time qPCR. All amplifications were performed using the QuantStudio™ 12K Flex system (Thermo Fisher, USA). The primers were produced by the company Exxtend (Brazil) presented in Table 1.

**Table 1:** PCR primers used for molecular detection of *H. pylori.*

Target	Primer PCR	Size (pb)
*ureA*	5' GAAGAAGCGAGAGCTGGTAAA 3'	116
	5' GGTATGCACGGTTACGAGTT 3'	
*16S*	5' GGACACACTGGAACTGAGAC 3'	162
	5' CCTAAAACCTTCATCCTCCAC 3'	
*cagA*	5' ACGCTCTGTCTTCTGTGTTAAT 3'	146
	5' CACATTGTTACCTTGTTGGTC 3'	

### Serological detection of the cagA factor

The detection of anti-cagA IgG antibodies in serum was performed using two commercial ELISA kits: the Human *Helicobacter pylori* cytotoxin-associated gene A protein IgG from MyBiosource^®^ (Canada) and the HP-cagA-IgG from Sunlong Biotech^®^ (China), following the manufacturer's specifications and conducted as a single assay (without duplicates). Blood samples with high levels of hemolysis and lipemia on visual inspection were excluded from the analyses. Briefly, microtiter plates pre-coated with recombinant CagA antigen were used to capture specific IgG antibodies present in serum samples. Serum samples and standards were diluted according to the protocol of both kits and incubated in the wells to allow binding between the anti-CagA IgG antibodies and the immobilized antigen. After washing to remove unbound components, a horseradish peroxidase (HRP)-conjugated secondary antibody targeting human IgG was added, followed by further incubation and washing steps.

Subsequently, a tetramethylbenzidine (TMB) substrate solution was added to each well. The enzyme-substrate reaction yielded a color change proportional to the concentration of anti-CagA IgG antibodies in the sample. The reaction was stopped by adding a sulfuric acid stop solution, and absorbance was measured at 450 nm using a microplate reader. Results were interpreted based on the cut-off values and criteria provided by each manufacturer. No meaningful differences were observed in the execution of the two tests evaluated. Samples were considered positive based on positive results from the rapid urease test, bacterial culture, and PCR tests for the *ureA* and *16S* genes. Thus, the results from the three biopsy-based tests were combined into a single variable that served as a reference for positivity.

### Statistical analysis

The performance of the commercial kits tested in adults was assessed by determining sensitivity, specificity, positive predictive value (PPV), and negative predictive value (NPV), with corresponding 95% confidence intervals. The agreement between the serological test results and those obtained from biopsy-based methods (culture, rapid urease test, and PCR) was determined by calculating the kappa coefficient and the McNemar chi-squared test.

To determine the optimal cutoff value for antibody IgG concentrations in predicting infection status in cagA-positive samples, the ROC (receiver operating characteristic) curve analysis was performed. The area under the ROC curve measures the assays' ability to discriminate between disease cases and healthy patients. An ideal test has a ROC curve area of 1. All statistical analyses were conducted using SPSS Statistics™ version 23.0.0.1 for Windows (IBM, USA), applying a significance level of 5%.

## Results

In this study, 88 individuals were recruited, revealing an *H. pylori* infection prevalence of 56.8% (50 cases). Among these positive cases, the prevalence of the cagA virulence factor was found to be 68%. Sociodemographic data related to the acquisition of the cagA virulence factor were determined through biopsy-based tests (Table 2). The validation study included 88 individuals, with 48 positive and 40 negative for *H. pylori*. In the group with positive cagA factor, 23 were women (67.6%), and 11 were men (32.4%). Among individuals who tested negative for the cagA factor, 29 were women (53.7%), and 25 were men (46.3%), with no significant difference in infection prevalence by sex (P-value=0.195). The mean age was 43 years, ranging from 16 to 75 years. Considering cagA infection status, the mean age was 42.3 years for positive cases and 45.4 years for negative cases.

**Table 2:** Sociodemographic characteristics of the sample.

Variables	Biopsy-based tests (n=88)		P- value
	Positive for the cagA factor (n=34)	Negative for the cagA factor (n=54)	
Gender			
Male	11 (32.4%)	25 (46.3%)	0.195
Female	23 (67.6%)	29 (53.7%)	
Age (mean±SD)	42.3±17.04	45.4±14.1	0.369
Ethnicity			
White	6 (17.6%)	14 (25.9%)	0.400
Asian yellow	1 (2.9%)	1 (1.9%)	
Black	27 (79.4%)	39 (72.2%)	
Education			
Illiterate - incomplete 1st grade	4 (11.8%)	11 (20.4%)	0.570
Complete primary - incomplete secondary	3 (8.8%)	(13.0%)	
Complete secondary - incomplete higher	13 (38.2%)	16 (29.6%)	
Not informed	0 (0.0%)	1 (1.9%)	
Higher education	14 (41.2%)	19 (35.2%)	
Marital status			
Married	20 (58.8%)	32 (59.3%)	0.717
Single	7 (20.6%)	15 (27.8%)	
Not informed	0 (0.0%)	2 (3.7%)	
Others	7 (20.6%)	5 (9.3%)	
Smoker/ex-smoker			
No	30 (88.2%)	45 (83.3%)	0.660
Yes	4 (11.8%)	9 (16.7%)	
Alcohol consumption			
No	20 (58.8%)	35 (64.8%)	0.341
Yes	15 (41.2%)	18 (33.3%)	
Family income			
<1 minimum wage	3 (8.8%)	3 (5.6%)	0.169
Between 1 and 2 salaries	9 (26.5%)	22 (40.7%)	
>2 salaries	22 (64.7%)	29 (53.7%)	
Relatives with gastric diseases			
No	26 (54.2%)	12 (30.0%)	0.223
Yes	22 (45.8%)	28 (70.0%)	
House water			
Treated water with internal plumbing	31 (91.2%)	49 (90.7%)	0.945
Others	3 (8.8%)	5 (9.3%)	
Sanitary waste			
Sewerage system	29 (85.3%)	45 (83.3%)	0.785
Closed septic tank	4 (11.8%)	8 (14.8%)	
Drainage network	0 (0.0%)	2 (3.7%)	
Stomach burn			
No	15 (44.1%)	21 (38.9%)	0.627
Yes	19 (55.9%)	33 (61.1%)	
Pain that causes night waking			
No	25 (73.5%)	32 (59.3%)	0.172
Yes	9 (26.5%)	22 (40.7%)	
Postprandial fullness			
No	12 (35.3%)	21 (38.9%)	0.743
Yes	22 (64.7%)	33 (61.1%)	
Stomach gas			
No	16 (47.1%)	22 (40.7%)	0.560
Yes	18 (52.9%)	32 (59.3%)	
Nausea			
No	23 (67.6%)	30 (55.6%)	0.259
Yes	11 (32.4%)	24 (44.4%)	
Vomit			
No	27 (79.4%)	44 (81.5%)	0.811
Yes	7 (20.6%)	10 (18.5%)	
Abdominal pain/location of pain			
No	20 (58.8%)	12 (22.2%)	0.268
Yes/epigastric and periumbilical	1 (2.9%)	4 (7.4%)	
Yes/epigastric	17 (50.0%)	28 (51.9%)	
Yes/periumbilical	1 (2.9%)	5 (9.3%)	

Data are reported as number and percentage. Chi-squared test.

Among the cagA + group, Black people were the most affected, accounting for 27 individuals of the positive cases (79.4%). They also represented the largest group among those who tested negative for the factor, with 39 individuals (72.2%). The educational status with the highest prevalence of positive cases for cagA was incomplete elementary education, accounting for 58.8% of the *H. pylori* cagA-positive individuals. Likewise, no statistically significant differences in the prevalence of the cagA factor were observed based on education level.

Regarding habits, four individuals were smokers or former smokers (11.8%), and there were no significant differences in cagA infection prevalence compared to non-smokers. Likewise, alcohol consumption had no significant association with *H. pylori* infection positive for cagA, with only 15 (41.2%) of the 33 individuals who consumed alcohol testing positive for the virulence factor. Married individuals were predominant in the cagA group, with 20 cases (58.8%) testing positive. Most active cases of *H. pylori* cagA+ occurred among individuals earning more than two minimum wages, totaling 22 positive cases (64.7%). This was followed by 9 cases (26.5%) in the income range of 1 to 2 minimum wages and 3 cases (8.8%) among those earning less than one minimum wage. The minority of patients had family history of gastric diseases and tested positive for the virulence factor (22 individuals, 45.8%). No significant associations were found between marital status, income, or family history of disease and the prevalence of cagA+ cases.

A large portion of the study population had access to piped water at home, with 31 positive cases (91.2%) and 49 negative cases (90.7%). Most also had access to sewage systems, with 29 positive (85.3%) and 45 negative (83.3%) for the cagA factor, showing no statistically significant difference in prevalence.

Infected and non-infected individuals presented gastric symptoms. Epigastric abdominal pain (50.0%), burning sensation (55.9%), postprandial fullness (64.7%), and abdominal distension (52.9%) were the symptoms with higher prevalence among *H. pylori* cagA+ patients. Night awakening (26.5%), nausea (32.4%), and vomiting (20.6%) occurred less frequently in individuals with cagA+ strains.

The ELISA kit assay from MyBiosource utilized a qualitative reverse-phase enzyme immunoassay technique. To determine the seropositivity and seronegativity of the samples, the mean of the negative controls (0.378) was added from the value specified by the kit (0.15) to establish the cutoff point, resulting in a value of 0.528. When the absorbance value was *<*cutoff, the sample was considered harmful for the cagA factor, and when the absorbance value was ≥cutoff, it was considered positive. The individual cutoff value was defined through each sample's absorbance value minus the established cutoff point (0.528). Table 3 shows the results of the statistical analyses of the test using the MedCalc statistical software calculator, with 95% confidence intervals.

**Table 3:** Statistical analysis considering the cutoff point of the MyBiosource cagA ELISA kit and the 95% confidence interval (95%CI) (95%).

Statistics	Value	95%CI
Sensitivity	20.59%	8.70 to 37.90%
Specificity	77.78%	64.40 to 87.96%
Positive likelihood ratio	0.93	0.40 to 2.12
Negative likelihood ratio	1.02	0.82 to 1.28
Disease prevalence*	38.64%	28.44 to 49.62%
Positive predictive value*	36.84%	20.32 to 57.16%
Negative predictive value*	60.87%	55.46 to 66.03%
Accuracy*	55.68%	44.70 to 66.27%

*These values are dependent on disease prevalence.

**Figure 1:**
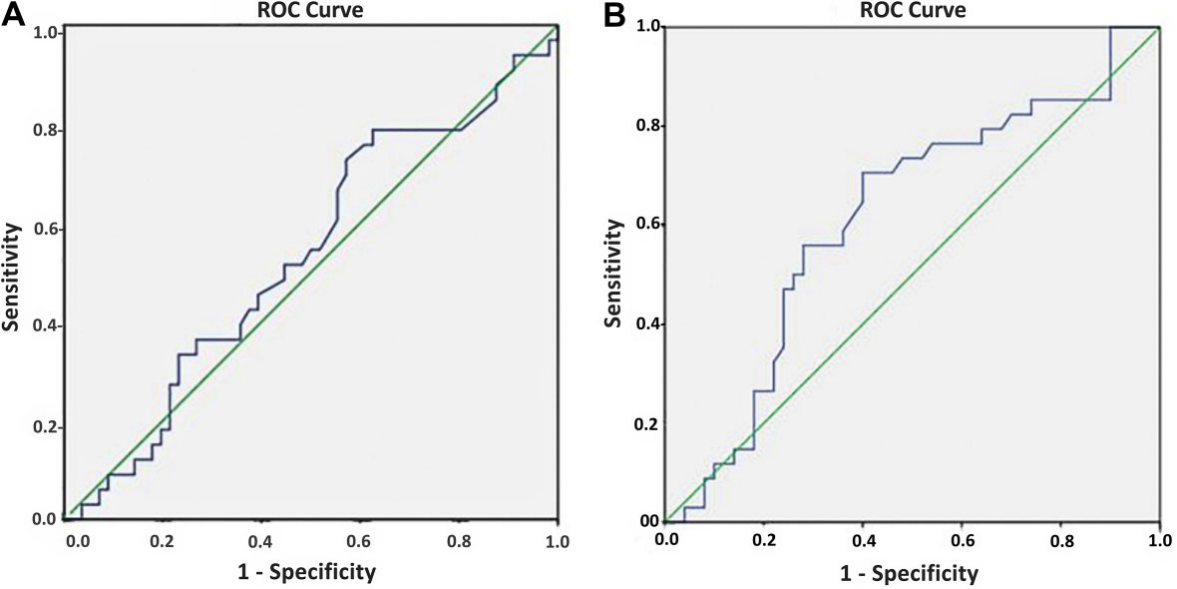
ROC curve for the cagA virulence factor using the (**A**) MyBiosource IgG cagA ELISA kit and the (**B**) Sunlong IgG cagA ELISA kit.

To improve the statistical results of the test, the ROC curve was used to define a new cutoff point to maximize the assay's sensitivity while decreasing the specificity within an acceptable limit (Figure 1A). The new cutoff point was defined as -0.21350, allowing the determination of new diagnostic test property values with corresponding 95% confidence intervals (Table 4). There was a significant increase in sensitivity from 20.59% in the initial analysis to 55.88%. Although specificity decreased from 77.78 to 48.15%, it remained within an acceptable confidence interval.

**Table 4:** Statistical analysis of the MyBiosource *cagA* ELISA kit, considering the new cutoff point (-0.21350) and the 95% confidence interval (95%CI).

Statistics	Value	95%CI
Sensitivity	55.88%	37.89 to 72.81%
Specificity	48.15%	34.34 to 62.16%
Positive likelihood ratio	1.08	0.73 to 1.60
Negative likelihood ratio	0.92	0.57 to 1.46
Disease prevalence*	38.64%	28.44 to 49.62%
Positive predictive value*	40.43%	31.39 to 50.16%
Negative predictive value*	63.41%	52.03 to 73.47%
Accuracy*	51.14%	40.25 to 61.95%

*These values are dependent on disease prevalence.

The Sunlong Biotech ELISA kit is known for its lack of a predefined method for determining the cutoff point despite being a quantitative assay designed to detect the cagA factor in picograms per milliliter (pg/mL). Subsequently, the cutoff value was established through ROC curve analysis, which indicated a cutoff of 82.093 pg/mL for the studied population (Figure 1B). The assay was executed as a quantitative analysis, employing the sandwich ELISA method with a pg/mL sensitivity of 1.

Absorbance values obtained from the samples were interpolated using the equation derived from the standard curve (360, 240, 120, 60, 30, and 0 pg/mL), comprising five calibration points and a blank. To ensure the precision of the assay, all absorbance measurements were adjusted to account for the blank value. The absorbance results exhibited a proportional relationship with the concentration of HP-cagA-IgG.

Table 5 shows the statistical analyses with corresponding 95% confidence intervals, considering the found cutoff point, indicating a sensitivity of 70.59% and specificity of 60.42%. The test identified 24 (29.3%) samples positive for the virulence factor.

**Table 5:** Statistical analysis of the Sunlong cagA ELISA Kit, considering the new cutoff point (82.093) and the 95% confidence interval (95%CI).

Statistics	Value	95%CI
Sensitivity	70.59%	52.52 to 84.90%
Specificity	60.42%	45.27 to 74.23%
Positive likelihood ratio	1.78	1.18 to 2.69
Negative likelihood ratio	0.49	0.28 to 0.86
Disease prevalence*	41.46%	30.68 to 52.88%
Positive predictive value*	55.81%	45.57 to 65.59%
Negative predictive value*	74.36%	62.15 to 83.67%
Accuracy*	64.63%	53.30 to 74.88%

*These values are dependent on disease prevalence.

Real-time PCR was the gold standard test to detect the cagA virulence factor. Of the 88 biopsy specimens, 34 (38.6%) were positive by PCR for the *cagA* gene (Table 6).

**Table 6:** Comparison of *H. pylori* cagA factor results by qPCR and serology.

Technique	*Helicobacter pylori* diagnosis n (%)			
	Positive	Negative	False positive	False negative
RT-PCR for *cagA* gene	34 (38.6%)	54 (61.4%)	0 (0.0%)	0 (0.0%)
Serology cagA-IgG Kit Sunlong	24 (29.3%)	29 (35.4%)	19 (23.2%)	10 (12.1%)
Serology cagA-IgG Kit MyBiosource	19 (21.6%)	26 (29.5%)	28 (31.8%)	15 (17.1%)

## Discussion

The present study detected a high prevalence of *cagA+* strains in the samples analyzed. The cagA protein is recognized as one of the most significant virulence factors of *H. pylori* and is strongly associated with gastric cancer development (7). In our study, real-time PCR, the gold standard for *cagA* detection, identified 34 positive cases (38.6%) among 88 participants. In comparison, a study of 159 patients reported a prevalence of cagA of 74.8% (14). In contrast, a more extensive study involving 751 individuals found that while 396 were positive for *H. pylori*, only 25 tested positive for the cagA factor, resulting in a lower prevalence rate of 25.5%.

Our results were in agreement with comparable studies from Brazil (15), Portugal (16), and Australia (17), which reported prevalence rates of 80.5, 61.7, and 66.7%, respectively, for the *cagA* gene. Conversely, lower rates of *cagA* detection have been reported in countries such as Egypt (35.7%) (17) and Chile (15.2%) (18). The variability in *cagA* gene prevalence is likely attributed to regional differences in *H. pylori* strains and infection rates (19).

A higher prevalence of *H. pylori cagA+* strains was observed among individuals aged 43. Although no significant difference was found between sexes (P=0.195), a more significant proportion of female participants was observed, with more women testing positive for *H. pylori cagA+.* While significant gender differences in infection acquisition have not been observed here, a higher rate in women has been previously reported (20,21).

No statistically significant association between race/ethnicity and cagA positivity was observed in the present study. However, studies conducted in the United States have reported a higher prevalence of *H. pylori cagA*+ infection among Black individuals and those of lower socioeconomic status (22,23). Stratification by educational level revealed that a higher proportion of individuals with higher education was present in the cagA+ (41.2%) and cagA- (35.2%) groups. This finding contrasts with previous studies, which suggest that lower education levels are more strongly associated with the development of *H. pylori* cagA+ (22,24). Given that this was a convenience sample drawn from a hospital in the private health sector, it is understandable that the population exhibited a higher level of education. Furthermore, the observed differences were not statistically significant.

Lifestyle factors such as smoking and alcohol consumption showed no significant association with *H. pylori cagA*+ infection in our study. While this finding aligns with similar studies (24-26), some authors have reported an increased risk among smokers and alcohol consumers (14,22). Income levels did not significantly differ between cases and controls, as both groups had a comparable proportion of individuals earning more than two minimum wages. This contrasts with findings from other studies, which suggest that higher income is associated with lower *H. pylori* infection rates (27,28). The higher prevalence of cases among individuals with higher income in our study may be attributed to participant recruitment from the private health sector, which serves a wealthier demographic. In contrast, the public Unified Health System (SUS) primarily caters to lower-income individuals.

No significant associations were observed between marital status and *H. pylori* cagA+ infection (P=0.717), corroborating findings from previous studies (26). Among participants positive for the cagA virulence factor, 91.2% had access to piped water, and 85.3% had sewerage systems in their homes. These figures were similar in the control group (90.7 and 83.3%, respectively) with no statistically significant difference. The lack of association between basic sanitation and *H. pylori* cagA+ infection contrasts with studies that have identified inadequate sanitation and lack of access to piped water as risk factors for *H. pylori* infection (3,29,30). The socioeconomic profile of our study population, which generally had good living conditions, may explain this discrepancy. Our study is the first to investigate the impact of sanitation on *H. pylori* cagA infection, contributing with novel insights on the subject.

No significant differences were found between cagA status and gastrointestinal symptoms, consistent with previous research (26). However, some studies have reported a correlation between cagA+ infection and symptoms such as stomach pain, burning sensation, vomiting, and nausea (15,24). In our study, cagA+ individuals exhibited prevalent symptoms, including burning sensation (55.9%), postprandial fullness (64.7%), and epigastric pain (50.0%). There was no significant association between symptoms and family history of *H. pylori cagA*+ infection.

The persistence of cagA antibodies even after a negative serological test for *H. pylori* highlights the complexity of interpreting infection status, as these antibodies may remain detectable long after the initial infection (31). Two ELISA kits were validated to assess the seroprevalence of anti-cagA IgG antibodies in serum, which is critical given the variability in test performance across different regions (32). This is due to the high level of genetic diversity and the genetic and antigenic differences among various strains and lineages of the bacteria (8,33). We identified 19 patients (21.6%) as cagA seropositive using the MyBiosource kit and 24 (29.3%) with the Sunlong Biotech kit.

The MyBioSource ELISA, designed for qualitative detection of cagA, showed suboptimal sensitivity (20.59%) and specificity (77.78%) in our population. ROC curve analysis allowed for the determination of a revised cutoff point, improving sensitivity to 55.88% and specificity to 48.15%. The Sunlong kit's quantitative assay did not provide a preset cutoff, necessitating validation through ROC analysis. This process yielded a sensitivity of 70.59% and a specificity of 60.42%.

This is the first study conducted in Southwestern Bahia to validate serological tests for the cagA virulence factor and analyze associated sociodemographic characteristics. Although new cutoff points improved sensitivity, both kits showed performance below 75%, indicating limited suitability for this study population. Additionally, serological tests may produce false negatives, particularly in early-stage infections (34). Although mild or moderate levels of hemolysis and lipemia in some of the samples used in the validation assays could negatively impact the performance of the evaluated kits, we believe that the impact of these pre-analytical factors - if any - was generally negligible and not enough to explain the poor performance observed. Despite limitations such as small sample size, reliance on a single qualitative and quantitative ELISA kit, and restricted recruitment, this study makes significant contributions. It paves the way for enhanced detection of antibodies to the cagA factor in the analyzed population, thereby improving its clinical utility and public health impact. Finally, considering the high cost of commercial serological kits currently available for detecting the cagA virulence factor of *H. pylori*, the results presented here may indicate that the development of in-house assays using *H. pylori* isolates from Brazilian populations may be a cheaper and more efficient and rational strategy. Such serological assays, more suited to the profile of our population, would be particularly useful for conducting population-based epidemiological studies that aim to identify genetic and socio-environmental factors related to the acquisition of cagA-positive *H. pylori* strains.

## Conclusion

The overall prevalence of *H. pylori* infection in the present study was low. However, the *cagA* gene was highly prevalent among patients who tested positive for the disease. The commercial tests did not perform satisfactorily regarding serological validation even after establishing a new cutoff point. Sensitivity remained below 75%, while specificity ranged between 50 and 60%. These results indicated that the development of customized (in-house) assays for the detection of anti-cagA IgG antibodies in our population may be a cheaper and feasible strategy compared to the currently available commercial assays.
